# A New Preoperative Scoring System for Predicting Aggressiveness of Non-Functioning Pancreatic Neuroendocrine Neoplasms

**DOI:** 10.3390/diagnostics12020397

**Published:** 2022-02-03

**Authors:** Tetsuya Takikawa, Kazuhiro Kikuta, Shin Hamada, Kiyoshi Kume, Shin Miura, Naoki Yoshida, Yu Tanaka, Ryotaro Matsumoto, Mio Ikeda, Fumiya Kataoka, Akira Sasaki, Hidehiro Hayashi, Waku Hatta, Yohei Ogata, Kei Nakagawa, Michiaki Unno, Atsushi Masamune

**Affiliations:** 1 Division of Gastroenterology, Tohoku University Graduate School of Medicine, Sendai 980-8574, Japan; t-takikawa@med.tohoku.ac.jp (T.T.); kkikuta@med.tohoku.ac.jp (K.K.); hamadas@med.tohoku.ac.jp (S.H.); kkume@med.tohoku.ac.jp (K.K.); miurashin@med.tohoku.ac.jp (S.M.); iiiiiiiiiiiiii14@hotmail.com (N.Y.); 1055@gmail.com (Y.T.); rmat44@gmail.com (R.M.); mio-0311@watch.ocn.ne.jp (M.I.); a9mb1039@yahoo.co.jp (F.K.); akira.ss.0911@gmail.com (A.S.); hayashisaporo@gmail.com (H.H.); waku-style@festa.ocn.ne.jp (W.H.); gmaps177@gmail.com (Y.O.); 2 Department of Surgery, Graduate School of Medicine, Tohoku University, Sendai 980-8574, Japan; kein@surg.med.tohoku.ac.jp (K.N.); m_unno@surg.med.tohoku.ac.jp (M.U.)

**Keywords:** endoscopic ultrasound-guided fine-needle aspiration, pancreatic cancer, pancreatic neuroendocrine neoplasm, prediction model, Ki-67

## Abstract

The management of non-functioning pancreatic neuroendocrine neoplasms (NF-PanNENs) is still controversial. This study aimed to develop a new scoring system for treatment decisions at initial diagnosis based on the identification of the predictive factors for aggressive NF-PanNENs. Seventy-seven patients who had been pathologically diagnosed with NF-PanNENs were enrolled. We retrospectively reviewed 13 variables that could be assessed preoperatively. Univariate and multivariate stepwise logistic regression analyses were performed to identify factors for the aggressiveness of NF-PanNENs, and a scoring system was developed by assigning weighted points proportional to their β regression coefficient. Tumor size > 20 mm on contrast-enhanced computed tomography, tumor non-vascularity, and Ki-67 labeling index ≥5% on endoscopic ultrasound-guided fine-needle aspiration specimens were identified as independent factors for predicting the aggressiveness of NF-PanNENs. The new scoring system, developed using the identified factors, had an excellent discrimination ability, with area under the curve of 0.92 (95% CI, 0.85–0.99), and good calibration (*p* = 0.72, Hosmer-Lemeshow test). Ten-year overall survival rates in low-risk (0 point), intermediate-risk (1 to 2 points), and high-risk (3 to 4 points) groups were 100%, 90.9%, and 24.3%, respectively. This new scoring system would be useful for treatment decisions and prognostic prediction at initial diagnosis.

## 1. Introduction

Pancreatic neuroendocrine neoplasms (PanNENs) are rare pancreatic tumors, accounting for 1–2% of all pancreatic tumors [1,2,3]. The number of PanNENs cases is steadily increasing [4,5,6]. The Surveillance, Epidemiology, and End Results (SEER) database revealed that the annual incidence of patients with PanNENs in the USA were 0.27 per 100,000 in the period 1987–1996, 0.43 in 1997–2006, and 1.01 in 2007–2016 [3]. The incease is particularly evident in asymptomatic and small non-functioning PanNENs (NF-PanNENs) [4,5,6]. According to a population-based study based in the USA, the incidence of pancreatic neuroendocrine tumors (PanNETs) ≤ 2 cm increased by 710% between 1988 and 2012 [4]. The proportion of the incidentally-detected cases increased from 19% of all NF-PanNENs cases in the period 2010–2011 to 57% in 2019–2020 [6]. These increases are likely to be attributable to the advances in imaging modalities, including endoscopic ultrasonography (EUS) [4,7]. EUS-guided fine-needle aspiration (EUS-FNA) has a high ability for histological diagnosis of PanNENs [7]. PanNENs were found in 0.8–10% of all autopsied cases [8,9], suggesting that the actual prevalence rates might be higher than those previously reported. Therefore, it is likely that the number of cases with PanNENs, particularly incidental and asymptomatic small lesions, might be further increased, and be of a more important clinical issue in the future.

Management of NF-PanNENs is still controversial. Tumor size is considered to be correlated with the aggressiveness of PanNENs [10,11,12], and recent studies have indicated that conservative management of small NF-PanNENs may be a good option based on their favorable prognosis [10,13,14,15]. The latest European Neuroendocrine Tumor Society (ENETS) guidelines proposed conservative management for NF-PanNENs with tumor size ≤ 20 mm in selected patients with significant comorbidity or advanced age [16]. The National Comprehensive Cancer Network (NCCN) guidelines for Neuroendocrine and Adrenal Tumors, version 4, 2021, proposed that observation can be considered for incidentally detected NF-PanNENs with tumor size ≤ 20 mm [17]. On the other hand, several studies have advocated that surgery should be performed even for small lesions ≤20 mm based on the improved survival rates compared with the conservative management group [4,18,19]. Because NF-PanNENs are biologically heterogenous [16], it may be difficult to accurately evaluate the aggressiveness of NF-PanNENs and to determine a management strategy solely based on tumor size. Other predictive factors for survival or recurrence after surgery include pathological lymph node metastasis, vascular invasion, tumor grade, and surgical resection margin [11,20,21,22,23]. Based on these predictive factors, several clinically relevant scoring systems have been developed [24,25,26,27]. However, these factors can be evaluated only after surgery using the resected specimens, and the development of scoring systems for treatment decisions at the initial diagnosis of NF-PanNENs is urgently needed. The aim of this study was to identify predictive factors for the aggressiveness of NF-PanNENs, which can be evaluated before surgery, and to develop a new scoring system for treatment decisions at initial diagnosis.

## 2. Materials and Methods

### 2.1. Study Design and Patients

This study was a single-center, retrospective, observational study. We analyzed the patients with PanNENs diagnosed at Tohoku University Hospital between June 2008 and December 2020. Inclusion criteria were as follows: (1) patients with pathologically diagnosed PanNENs by surgical or EUS-FNA specimens; and (2) patients who had undergone preoperative EUS-FNA and multiphase contrast-enhanced computed tomography (CE-CT) at initial diagnosis. Exclusion criteria were as follows: (1) functioning PanNENs; (2) hereditary diseases such as multiple endocrine neoplasia type 1 (MEN-1) and von Hippel–Lindau (VHL) diseases; (3) presence of synchronous malignancies; (4) insufficient EUS-FNA sample for pathological evaluation; (5) undetectable tumors on CE-CT; and (6) less than 6 months of follow-up period.

### 2.2. Data Collection and Candidate Variables

Clinical, imaging, and pathological data were collected from medical records. We reviewed age, sex, and presence of symptoms as clinical variables. We selected additional candidate variables based on the previous studies showing their utilities for the prediction of aggressive PanNENs. We evaluated the tumor size, tumor location, number of tumors, tumor vascularity, cystic degeneration/necrosis, calcification, and main pancreatic duct (MPD) and common bile duct (CBD) involvement on CE-CT [28,29,30,31,32]. Abnormal uptake on ^18^F-fluorodeoxyglucose (^18^F-FDG) positron emission tomography (PET)/CT was also assessed, because its utility to predict prognosis and tumor grade has been reported in patients with PanNENs [33]. We included Ki-67 labeling index (LI) on EUS-FNA specimens as another candidate variable [34,35]. Regarding its threshold, the World Health Organization (WHO) classification define Ki-67 LI ≥ 3% as the threshold for tumor grading [36], but recent studies have reported that Ki-67 LI ≥ 5% was a good threshold of aggressiveness [37,38]. Therefore, we analyzed Ki-67 LI of both 3% and 5% on EUS-FNA specimens as pathological variables. We did not include ^68^Ga-DOTA-Tyr^3^-octreotide PET because it is not covered by medical insurance in Japan and no patients underwent the examination.

### 2.3. Definitions

NF tumors were defined as tumors with no symptoms of hormonal excess regardless of the laboratory data. We determined the tumor grade according to the WHO 2017 classification [36] based on Ki-67 LI and mitotic count using surgical specimens in resected cases and on Ki-67 LI using EUS-FNA specimens in non-resected cases. We used the ENETS TNM staging system [39] for disease stage classification. We defined aggressive NF-PanNENs as disease-related death, recurrence after surgery, pathological and imaging lymph node metastases, and distant metastases at initial diagnosis according to the previous reports with some modifications [40,41].

Tumor size was defined as the largest diameter measured on CE-CT at initial diagnosis. To evaluate the vascularity, we measured the Hounsfield units (HU) value by placing the oval region of interest (ROI) of 10 mm^2^ within the tumor in arterial phase on CE-CT, avoiding areas of calcification, cystic degeneration/necrosis, and pancreatic duct [32,42]. Hypervascular tumor was defined as if the HU value in the arterial phase within the tumor was at least 10 HU higher than that of the surrounding normal pancreatic parenchyma [32]. Cystic degeneration/necrosis was defined as non-enhanced areas of circular or ovoid shape, and the calcification within the tumor was assessed on plain CT image [42]. MPD involvement was defined as interruption of the MPD with upstream dilatation (≥3 mm) [29,30]. CBD involvement was defined as interruption of the CBD with upstream dilatation (≥10 mm) or symptoms of jaundice [30]. Imaging lymph node metastases were diagnosed when there was a diameter ≥10 mm with irregular margins or heterogeneous enhancement, when there was an abnormal uptake on somatostatin receptor scintigraphy (SRS) [28].

Significant progression was defined as a greater than 5 mm or 20% increase in total in the size of the primary tumor from the baseline [43]. Overall survival (OS) was defined as the time from pathological diagnosis to the date of the last follow-up or death due to any cause. The definition of disease-free survival (DFS) was the period from surgical resection to radiological evidence of local recurrence, distant metastasis, or death due to any cause.

### 2.4. Statistical Analysis

Continuous variables were presented as mean (standard deviation (SD)) or median (interquartile range (IQR)), and categorical variables were expressed as numbers (percentages). For comparison between two groups, Student’s t-test or Wilcoxon rank sum test was used for continuous variables, and the chi-square test or Fisher’s exact test was used for categorical variables. Survival curves were estimated using the Kaplan–Meier method and compared using the Log-rank test.

For development of a scoring system, we performed a univariate and multivariate stepwise logistic regression analyses. Candidate variables with a difference of *p* < 0.2 in univariate analysis were entered into forward stepwise selection based on Akaike’s Information Criterion (AIC), and selected variables were analyzed by multivariate analysis. We allocated points proportional to β regression coefficient values for the predictive variables determined in the multivariate analysis as previously reported [44,45]. The coefficient of each variable was divided by the lowest β value among variables included into the final prediction model and rounded to the nearest integer. The adjustment of each coefficient is a standard method for driving a scoring system [46]. The total score in each patient was then calculated. The model’s discrimination was assessed by the area under the curve (AUC) in the receiver operating characteristic curve, and its calibration was evaluated by the Hosmer–Lemeshow test. Internal validation was estimated using the bootstrap resampling with 1000 repetitions. The Cochran–Armitage test was used to analyze trends in risk groups according to the scoring system. The AUC values were compared using the DeLong test [47].

JMP Pro 16 (SAS Institute Inc., Cary, NC, USA), IBM SPSS Statistics 21 (IBM Corp., Armonk, NY, USA), and R version 3.6.1 for Windows software (R Foundation) were used for statistical analysis, and a two-sided *p*-value < 0.05 was considered statistically significant.

## 3. Results

### 3.1. Characeteristics of the Enrolled Patients

**Figure 1:**
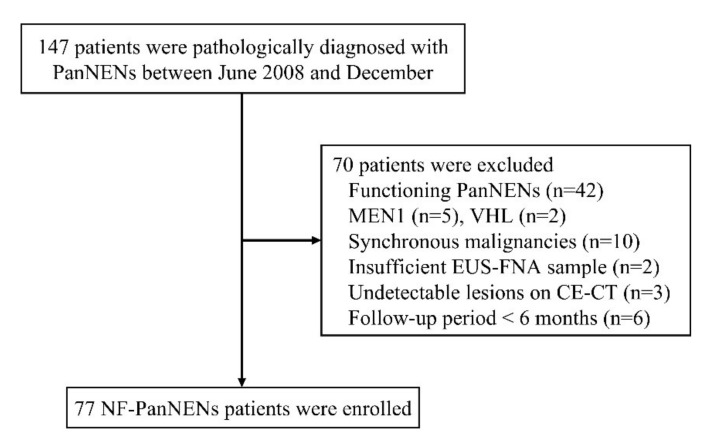
Flowchart of patient enrollment.

Figure 1 shows the flowchart of patient enrollment. During the study period, 147 patients met the inclusion criteria. The following patients were excluded: those with functioning PanNENs (*n* = 42), hereditary diseases such as MEN-1 (*n* = 5) and VHL (*n* = 2), synchronous other malignancies (*n* = 10), insufficient EUS-FNA sample for pathological evaluation (*n* = 2), undetectable lesions on CE-CT (*n* = 3), less than 6 months of follow-up period (*n* = 6). Finally, 77 patients with NF-PanNENs were enrolled, and their clinicopathological characteristics are shown in Table 1. The mean patient age (SD) was 61.1 (12.9) years, and 38 (49.4%) patients were male. The median tumor size (IQR) at initial diagnosis was 18 (12–34) mm. Lymph node and distant metastases occurred in 19 patients (24.7%) and 16 patients (20.8%), respectively. The tumor grades, based on the WHO 2017 classification, were G1 for 38 patients (49.4%), G2 for 26 (33.8%), NET G3 for 3 (3.9%), and neuroendocrine carcinoma (NEC) G3 for 10 (13.0%). Fifty-four patients (70.1%) underwent surgical resection immediately after the diagnosis, 14 (18.2%) received chemotherapy, and 8 (10.4%) underwent follow-up surveillance. The median follow-up period (IQR) was 1636 (568–3024) days. Five-year and ten-year OS rates were 85.9% and 76.3%, respectively. For the 8 patients who underwent follow-up surveillance, the mean patient age (SD) was 70.5 (9.0) years, and 7 (87.5%) were female. The median tumor size (IQR) at initial diagnosis was 9 (7–13) mm. Significant progression was not observed in any of these 8 patients during the median observation period of 1621 days.

Of the 77 patients, 46 (59.7%) were classified into the non-aggressive group and 31 (40.3%) were classified into the aggressive group. Table 2 shows the comparison of baseline characteristics between the two groups. There were significant differences in tumor size (*p* < 0.001), tumor grade (*p* < 0.001), disease stage (*p* < 0.001), treatment (*p* < 0.001), and prognosis (*p* < 0.001) between the two groups. There were 50 patients whose tumor size was ≤20 mm. Among them, 9 (18%) patients were classified into the aggressive group due to lymph node metastasis (*n* = 3), distant metastasis (*n* = 2), postoperative recurrence (*n* = 3), or disease-related death due to distant metastasis (*n* = 1). In the non-aggressive group, G1 tumors accounted for 76.1%, and no G3 tumors were observed. As for the ENETS stage, 87.0% of the patients in the non-aggressive group were Stage I, whereas approximately 80% in the aggressive group were Stage III or IV. The 5-year and 10-year OS rates were both 96.8% in the non-aggressive group, and 70.0% and 50.9% in the aggressive group, respectively. The median follow-up period in the non-aggressive and aggressive groups were 1655 and 1395 days, with no significant difference (*p* = 0.19).

### 3.2. Univariate and Multivariate Stepwise Logistic Regression Analyses of Candidate Variables

We performed univariate logistic regression analysis to select candidate variables to predict the aggressiveness of NF-PanNENs, and age (*p* = 0.09), presence of symptoms (*p* < 0.001), tumor size (*p* < 0.001), tumor vascularity (*p* < 0.001), cystic degeneration/necrosis (*p* = 0.002), tumor calcification (*p* = 0.10), MPD or CBD involvement (*p* = 0.003), abnormal uptake on ^18^F-FDG PET/CT (*p* = 0.003), and EUS-FNA Ki-67 LI ≥ 3% (*p* < 0.001) and ≥ 5% (*p* < 0.001) revealed *p* value of <0.2 (Table 3). Among them, three variables (tumor size, tumor vascularity, and EUS-FNA Ki-67 LI ≥ 5%) were selected by forward stepwise selection based on the Akaike’s Information Criterion. Multivariate logistic regression analysis revealed that tumor size > 20 mm (*p* = 0.004), tumor non-hypervascularity (*p* = 0.001), and EUS-FNA Ki-67 LI ≥ 5% (*p* = 0.034) were independently associated with the aggressiveness of NF-PanNENs (Table 4).

### 3.3. Development of a Scoring System

We then developed a scoring system using the three identified factors based on their β regression coefficient values. Because the lowest coefficient value was 1.94 for EUS-FNA ki-67 LI, the β regression coefficient values of each factor were divided by 1.94 and rounded to the nearest integer. As a result, tumor size > 20 mm was assigned as 1 point, tumor non-hypervascularity as 2 points, and EUS-FNA Ki-67 LI ≥ 5 % as 1 point (Table 4). The discrimination ability of the model was excellent, with AUC of 0.92 (95% confidence interval (CI), 0.85–0.99) with standard error (SE) of 0.04. Based on the cut-off level of 2 points, the positive and negative predictive values were 88.9% and 86.0%, respectively. The Hosmer–Lemeshow test also indicated good calibration (*p* = 0.72). The model was internally validated using bootstrap resampling with 1000 repetitions, which showed mean AUC of 0.92 (95% CI, 0.84–0.98) with SE of 0.001.

**Figure 2:**
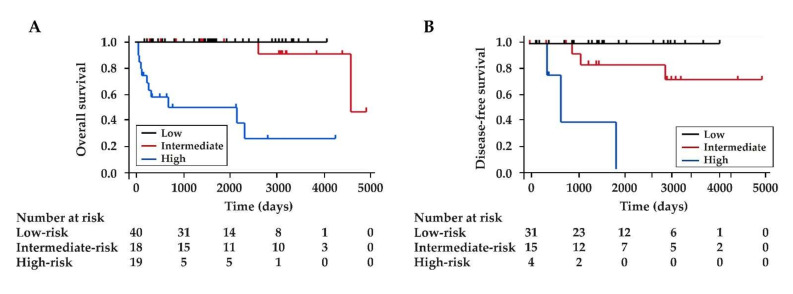
Kaplan–Meier survival curves of overall and disease-free survival stratified by risk groups. (**A**) Overall survival of all enrolled patients and (**B**) disease-free survival of the patients who underwent R0 resection were different between the three groups (both for *p* < 0.001).

We stratified the patients into three risk groups according to the total points: low-risk (0 point), intermediate-risk (1 to 2 points), and high-risk (3 to 4 points). The proportions of patients with aggressive NF-PanNENs in the low-risk, intermediate-risk, and high-risk groups were 7.5%, 50.0%, 100%, respectively (Table 5 and Table 6). There was an increasing trend from the low-risk to the high-risk groups (*p* < 0.001, Cochran–Armitage trend test). The 1-year, 5-year, and 10-year OS rates were different between the three groups (*p* < 0.001, Log-rank test): all 100% for the low-risk group; 100%, 92.9%, and 84.4% for the intermediate-risk group; and 56.7%, 48.6%, and 24.3% for the high-risk group, respectively (Figure 2A). Of the 50 patients who underwent R0 resection, the 1-year, 5-year, and 10-year DFS rates were different between the three groups (*p* < 0.001): all 100% for the low-risk group; 100%, 71.4%, and 71.4% for the intermediate-risk group; and 75.0%, 0%, and 0% for the high-risk group, respectively (Figure 2B).

### 3.4. The Comparison between the New Scoring System and ENETS TNM Staging System

**Figure 3:**
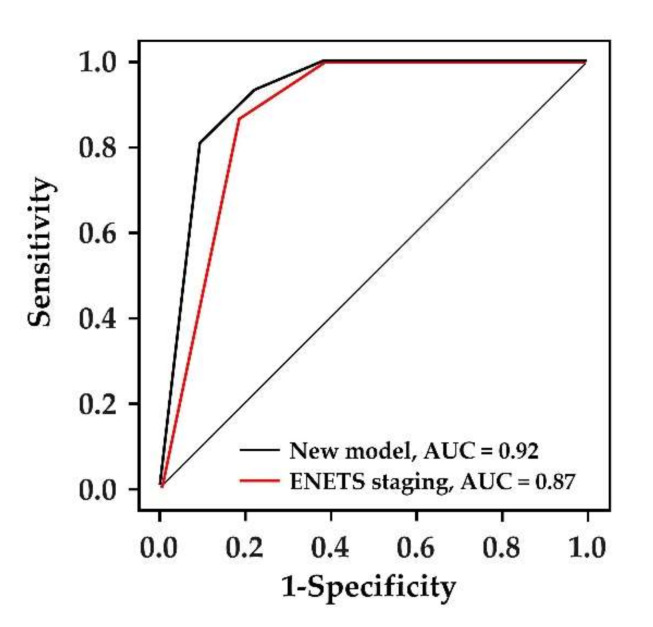
Receiver operating characteristic curve for predicting aggressiveness of non-functioning pancreatic neuroendocrine neoplasms. Although it was not statistically significant, the new model had a higher value of area under the curve than the ENETS staging system (*p* = 0.13).

We compared the model performance to predict the aggressiveness of NF-PanNENs between the new scoring system and the ENETS TNM staging system. The AUC of the new scoring model and ENETS TNM staging system were 0.92 (95% CI, 0.85–0.99) with SE of 0.04 and 0.87 (95% CI, 0.77–0.94) with SE of 0.05, respectively. Although it was not statistically significant, the new model had a higher value of AUC than the ENETS staging system (*p* = 0.13, DeLong test) (Figure 3).

## 4. Discussion

In this study, we developed a new scoring system useful for treatment decisions at initial diagnosis in patients with NF-PanNENs. We first identified the predictive factors for the aggressiveness of NF-PanNENs, which can be evaluated preoperatively without resected specimens. Two imaging factors on CE-CT (tumor size > 20 mm and tumor non-hypervascularity) and one pathological factor (Ki-67 LI ≥ 5% on EUS-FNA specimens) were identified as independent factors associated with the aggressiveness of NF-PanNENs. We then developed a scoring system using these three identified factors. These factors were not included in our definition of aggressive NF-PanNENs. This new scoring system had a reliable performance and could stratify the long-term prognosis and the postoperative recurrence. The strength of this new scoring system is that it can be evaluated before treatment, because the resected specimens were not required for the evaluation. This scoring system may provide useful information for treatment decisions, such as surgery and surveillance, and predicting the prognosis in patients with NF-PanNENs.

We identified tumor size as one of the predictive factors. This agrees with the previous studies showing that tumor size was associated with tumor grade, prognosis, and postoperative recurrence [10,11,12]. Although 20 mm is a previously reported cut-off value for distinguishing malignancy [16,17], highly malignant lesions might exist in tumors ≤ 20 mm [19,31]. Millis et al. [19] reported that 38% (24/66) of sporadic NF-PanNENs with tumors ≤ 20 mm had malignant features such as vascular invasion, lymph node, and distant metastases. In a multicenter study of 210 resected NF-PanNENs cases with tumors ≤ 20 mm, Sallinen et al. [31] reported that 10.6% of cases had lymph node metastases and 19% had G2–3 tumors. In this study, 9/50 (18%) patients whose tumor size was ≤20 mm were classified into the aggressive group. Collectively, tumor size alone is insufficient for estimating the aggressiveness of NF-PanNENs.

In addition to tumor size, we identified the non-hypervascularity of tumors as an independent predictive factor. Tumor non-hypervascularity had the highest β regression coefficient among the three identified factors and was given the highest point in the scoring system. Our result supports the previous studies showing the association of non-vascularity with tumor grade [30,48]. Zamboni et al. [48] reviewed 154 patients with NF-PanNENs and reported that arterial vascularization differentiated tumor grade, with G1 tumors being more hypervascular and G3 tumors being more non-hypervascular. Yamada et al. [30] analyzed 37 NF-PanNENs of G1 and G2 with preoperative multiphase CT and reported that HU value in G1 tumors was higher than that of G2 tumors. They also reported the AUC of HU value was higher than that of tumor size. On the other hand, in a multicenter retrospective study of 158 patients with surgically resected NF-PanNENs ≤ 20 mm [49], hyperenhancement in the arteria phase was not associated with metastases or recurrences. However, this study included only small lesions, and 87% of patients were diagnosed by surgical specimens, indicating that the patients with high malignant potential such as unrespectable cases might have been excluded. In our study, 56.5% (13/23) of non-hypervascular NF-PanNENs had distant metastases at initial diagnosis (data not shown). Non-hyperenhancement suggests aggressive lesions.

Recently, there is accumulating evidence that EUS is useful for predicting the aggressiveness of PanNENs [9,41,50]. For example, Crinó et al. [50] showed irregular margins and tumor size > 20 mm on EUS were associated with malignancy and aggressiveness of PanNENs. Ishikawa et al. [41] showed hypo-enhancement on contrast-enhanced harmonic EUS (CE-EUS) was an indicator of aggressive PanNENs. Compared to CE-CT, CE-EUS has advantages such as a higher detection rate of small lesions and the ability to use contrast media even in patients with renal failure. Further studies are needed to clarify whether CE-EUS is superior to CE-CT for predicting malignancy, or whether a combination of the two modalities increases diagnostic abilities.

In general, tumor grade based on Ki-67 LI strongly reflects the malignant potential of NF-PanNENs [16,36], and Ki-67 LI on EUS-FNA specimens has been reported as a useful prognostic factor. Boutsen et al. [34] reported that tumor grade on EUS-FNA specimens was associated with OS in 101 patients with NF-PanNENs. Cui et al. [35] reported similar results in 37 resected cases. However, it is known that Ki-67 LI in EUS-FNA specimens is often inconsistent with that obtained on surgical specimens [7,51]. A pooled analysis showed a grade concordance rate of 77.5% between EUS-FNA and surgical specimens [7]. Importantly, previous studies showed a better tumor grade concordance rate between EUS-FNA and surgical specimens in smaller PanNENs [52,53]. Paiella et al. [53] reported that correlation of Ki-67 LI between EUS-FNA and surgical specimens was strong in PanNENs ≤ 20 mm, whereas it was moderate in PanNENs > 20 mm. A EUS-guided fine needle biopsy (EUS-FNB) sampling procedure, using new core biopsy needles, has been developed to improve the sample quantity and quality [54]. A meta-analysis of 11 randomized controlled trials revealed that EUS-FNB had a better specimen adequacy, higher diagnostic accuracy, and a fewer number of needle-passes than EUS-FNA for sampling pancreatic masses [54]. This might also be the case for evaluation of the Ki-67 LI in PanNENs. Crinó et al. [55] reported that EUS-FNB specimens had a stronger correlation with surgical specimens for Ki-67 LI than EUS-FNA. They also showed that EUS-FNB specimens had better assessment feasibility of Ki-67 LI than EUS-FNA specimens in PanNETs ≤ 20 mm. Ki-67 LI assessment by EUS-FNA/B is essential for preoperative prediction for the aggressiveness of NF-PanNENs, and the widespread use of EUS-FNB would increase the reliability of the present scoring system, especially for lesions ≤20 mm.

Although previous studies have developed scoring models for predicting the malignant potential of NF-PanNENs, most of them could be evaluated only after surgery [24,25,26,27]. Fisher et al. [56] reported a risk score focusing on preoperative factors, including chromogranin A, tumor grade, tumor size, and presence of metastasis. However, they reviewed patients with curative resection, indicating that the patients with high malignancy were not included. In addition, tumor grade was evaluated based on final pathological report, therefore their model could not be used for preoperative treatment decisions. Primavesi et al. [57] developed a scoring model using only preoperative factors, including C-reactive protein, presence of metastasis, and tumor size, which were associated with the prognosis of NF-PanNENs patients. However, their study included only patients with curative resection, and postoperative factors, such as tumor grade and lymph node metastasis based on surgical specimens, were included. Unlike these previous studies, we employed predictable factors available preoperatively, and we analyzed all of the resected, unresectable, and surveillance cases. Our scoring system had a high discrimination ability (AUC, 0.92). The assessment of tumor vascularity on CE-CT and pathological evaluation of Ki-67 LI on EUS-FNA specimens, which are not included in the ENETS TNM staging system, may contribute to the increased discrimination ability.

The usefulness of less-invasive EUS-guided radiofrequency ablation (EUS-RFA) has been recently reported [58]. EUS-RFA might be a treatment option for patients in the low-risk group, particularly for those with high surgical risk. EUS-RFA is not basically indicated for patients in the intermediate-risk group because 33.3% (6/18) had lymph node metastasis (data not shown). Therefore, for patients in the intermediate- and high-risk groups, surgery should be selected if the patient can tolerate it. Neoadjuvant chemotherapy might be considered in the future, because all patients with R0 resection in these groups showed postoperative recurrence within 5 years.

This study has several limitations. First, the sample size is relatively small and external validation is lacking, which may lead to overestimation of the new scoring system. Although events per variable in this study (31/3 = 10.3) met the recommended amount (over 10) to fit a prediction model using logistic regression [59], the small sample size led to a wide 95% CI in each variable. Second, this study was a single-center and retrospective study. Third, this scoring model requires lesions detectable on CE-CT and sufficient samples on EUS-FNA specimens. Due to this limitation, we excluded two patients who were undetectable on CE-CT and three patients with insufficient samples by EUS-FNA. Recent studies have demonstrated that NF-PanNENs ≤10 mm had very low malignant potential [15,49]. The scoring system might require further stratification based on tumor size ≤ 10 mm, which could not be performed due to the small sample size in this study.

## 5. Conclusions

We here developed a new scoring system for predicting the aggressiveness of NF-PanNENs using the three factors (tumor size, tumor non-hypervascularity on CE-CT, and Ki-67 LI on EUS-FNA specimens) that can be assessed preoperatively. This model may be useful for treatment decisions as well as for prognosis prediction at initial diagnosis. Further multi-center, prospective studies are warranted to validate this scoring system in larger cohorts.

## Figures and Tables

**Table 1:** Clinicopathological characteristics of patients with NF-PanNENs.

Variables	NF-PanNENs (*n* = 77)
Age, mean (SD), years	61.1 (12.9)
Sex, male, *n* (%)	38 (49.4)
Median tumor size, mm (IQR)	18 (12–34)
Symptoms, yes, *n* (%)	26 (33.8)
Tumor location, *n* (%)	
Head	30 (39.0)
Body/Tail	44 (57.1)
Multiple	3 (3.9)
Lymph node metastasis, *n* (%)	19 (24.7)
Distant metastasis, *n* (%)	16 (20.8)
Tumor grade (WHO 2017), *n* (%)	
G1	38 (49.4)
G2	26 (33.8)
NET G3	3 (3.9)
NEC G3	10 (13.0)
ENETS Stage, *n* (%)	
I	42 (54.5)
II	11 (14.3)
III	8 (10.4)
IV	16 (20.8)
Treatment, *n* (%)	
Surgery	54 (70.1)
Chemotherapy	14 (18.2)
Surveillance	8 (10.4)
Best supportive care	1 (1.3)
Overall survival rate (%)	
5-year OS rate	85.9
10-year OS rate	76.3
Median follow-up period, days (IQR)	1636 (568–3024)

NF-PanNENs, non-functioning pancreatic neuroendocrine neoplasms; SD, standard deviation; IQR, interquartile range; WHO, World Health Organization; NET, neuroendocrine tumor; NEC, neuroendocrine carcinoma; ENETS, European Neuroendocrine Tumor Society; OS, overall survival.

**Table 2:** Baseline characteristics between the non-aggressive and aggressive groups.

Variables	Non-Aggressive Group (*n* = 46)	Aggressive Group (*n* = 31)	*p* Value
Age, mean (SD), years	63.3 (11.5)	57.9 (14.3)	0.07
Sex, male, *n* (%)	22 (47.8)	16 (51.6)	0.74
Median tumor size, mm (IQR)	14 (9–18)	38 (20–53)	<0.001
Tumor grade (WHO 2017), *n* (%)			<0.001
G1	35 (76.1)	3 (9.7)	
G2	11 (23.9)	15 (48.4)	
NET G3	0 (0)	3 (9.7)	
NEC G3	0 (0)	10 (32.3)	
ENETS Stage, *n* (%)			<0.001
I	40 (87.0)	2 (6.5)	
II	6 (13.0)	5 (16.1)	
III	0 (0)	8 (25.8)	
IV	0 (0)	16 (51.6)	
Treatment, *n* (%)			<0.001
Surgery	37 (80.4)	17 (54.8)	
Chemotherapy	1 (2.2)	13 (41.9)	
Surveillance	8 (17.4)	0 (0)	
Best supportive care	0 (0)	1 (3.2)	
Prognosis, (%)			<0.001
5-year OS rate	96.8	70.0	
10-year OS rate	96.8	50.9	
Median follow-up period, days (IQR)	1655 (287–2824)	1395 (865–3055)	0.19

SD, standard deviation; IQR, interquartile range; WHO, World Health Organization; NET, neuroendocrine tumor; NEC, neuroendocrine carcinoma; ENETS, European Neuroendocrine Tumor Society; OS, overall survival.

**Table 3:** Univariate analysis of candidate variables.

Variables	Non-Aggressive Group (*n* = 46)	Aggressive Group (*n* = 31)	OR (95% CI)	*p* Value
Age (years), *n* (%)				0.09
<65	22 (47.8)	21 (67.7)	1	
≥65	24 (52.2)	10 (32.3)	0.44 (0.09–1.13)	
Sex, *n* (%)				0.74
Female	24 (52.2)	15 (48.4)	1	
Male	22 (47.8)	16 (51.6)	1.16 (0.47–2.90)	
Symptoms, *n* (%)				<0.001
No	40 (87.0)	11 (35.5)	1	
Yes	6 (13.0)	20 (64.5)	12.12 (3.91–37.53)	
Tumor location, *n* (%)				0.65
Head	16 (34.8)	14 (45.2)	1	
Body/tail	28 (60.9)	16 (51.6)	0.65 (0.60–3.94)	
Multiple	2 (4.3)	1 (3.2)	0.57 (0.05–7.00)	
Number of tumors, *n* (%)				0.80
Single	44 (95.7)	30 (96.8)	1	
Multiple	2 (4.3)	1 (3.2)	0.73 (0.06–8.45)	
Tumor size (mm), *n* (%)				<0.001
≤20	41 (89.1)	9 (29.0)	1	
>20	5 (10.9)	22 (71.0)	20.0 (5.98–67.20)	
Tumor vascularity, *n* (%)				<0.001
Hypervascular	44 (95.7)	10 (32.3)	1	
Non-hypervascular	2 (4.3)	21 (67.7)	46.2 (9.28–229.91)	
Cystic degeneration/necrosis, *n* (%)				0.002
No	37 (80.4)	14 (45.2)	1	
Yes	9 (19.6)	17 (54.8)	1.81 (1.81–13.78)	
Tumor calcification, *n* (%)				0.10
No	45 (97.8)	27 (87.1)	1	
Yes	1 (2.2)	4 (12.9)	6.67 (0.71–62.79)	
MPD or CBD involvement, *n* (%)				0.003
No	43 (93.5)	20 (64.5)	1	
Yes	3 (6.5)	11 (35.5)	7.88 (1.98–31.41)	
^18^F-FDG PET/CT ^#^, *n* (%)				0.003
Negative	23 (54.8)	5 (17.9)	1	
Positive	19 (45.2)	23 (82.1)	5.57 (1.78–17.45)	
EUS-FNA Ki-67 LI ≥ 3%, *n* (%)				<0.001
No	38 (82.6)	9 (29.0)	1	
Yes	8 (17.4)	22 (71.0)	11.61 (3.91–34.45)	
EUS-FNA Ki-67 LI ≥ 5%, *n* (%)				<0.001
No	43 (93.5)	11 (35.5)	1	
Yes	3 (6.5)	20 (64.5)	26.06 (6.54–103.84)	

OR, odds ratio; CI, confidence interval; MPD, main pancreatic duct; CBD, common bile duct; ^18^F-FDG PET/CT, ^18^F-fluorodeoxyglucose positron emission tomography/computed tomography; EUS-FNA, endoscopic ultrasound-guided fine-needle aspiration; LI, labeling index. ^#^ Excluding 7 patients (4 in the non-aggressive and 3 in the aggressive groups) who did not undergo ^18^F-FDG PET/CT.

**Table 4:** Multivariate stepwise logistic regression analysis of selected variables.

Variables		OR (95% CI)	*p* Value	β Regression Coefficients	SE	Points
Tumor size	>20 mm	9.96 (2.05–48.46)	0.004	2.30	0.80	1
Tumor vascularity	Non-hypervascular	23.23 (3.54–152.44)	0.001	3.15	0.96	2
EUS-FNA Ki-67 LI	≥5%	6.95 (1.16–41.80)	0.034	1.94	0.92	1

OR, odds ratio; CI, confidence interval; SE, standard error; EUS-FNA, endoscopic ultrasound-guided fine-needle aspiration; LI, labeling index.

**Table 5:** The proportions of aggressive NF-PanNENs according to the total points.

Total Points	Patients, *n*	Proportions of Aggressive NF-PanNENs, %
0	40	7.5 (3/40)
1	10	40.0 (4/10)
2	8	62.5 (5/8)
3	6	100 (6/6)
4	13	100 (13/13)

**Table 6:** Proportions of aggressive NF-PanNENs according to the risk groups.

Risk Groups	Total Points	Patients, *n*	Proportions of Aggressive NF-PanNENs, %
Low-risk	0	40	7.5 (3/40)
Intermediate-risk	1 to 2	18	50.0 (9/18)
High-risk	3 to 4	19	100 (19/19)

NF-PanNENs, non-functioning pancreatic neuroendocrine neoplasms.

## Data Availability

The data used during the current study are available from the corresponding author upon reasonable request.
